# The Effects of Immunosuppressive Factors on Primary Dendritic Cells from C57BL/6 and CBA Mice

**DOI:** 10.1155/2019/7029726

**Published:** 2019-04-18

**Authors:** Vasiliy V. Kurilin, Julia N. Khantakova, Valeriy P. Tereschenko, Julia A. Lopatnikova, Irina A. Obleukhova, Sergey V. Sennikov

**Affiliations:** ^1^Laboratory of Molecular Immunology, Federal State Budgetary Institution “Research Institute of Fundamental and Clinical Immunology”, Yadrintsevskaya St. 14, Novosibirsk, 630099, Russia; ^2^Novosibirsk State University, Pirogova St. 2, Novosibirsk, 630090, Russia

## Abstract

**Introduction:**

Dendritic cells (DCs) control immune responses by modulating T and B cells towards effector or tolerogenic responses. In this study, we evaluated the effects of different immunosuppressive molecules on the phenotypic and functional characteristics of primary dendritic cells from C57BL/6 and CBA mice.

**Methods:**

DCs were derived from bone marrow cells in the presence of rmGM-CSF and rmIL-4. DCs were then treated with different types of immunosuppressive molecules (rmIL-10, rmTGF-*β*, and BAY 11-7082) and cocultured with syngeneic splenocytes. The amount of CD4+CD25hiFoxP3+ Tregs, IL-10 expression, and proliferation were evaluated.

**Results:**

Tolerogenic factors were found to have different effects on DCs C57Bl/6 mice. In C57Bl/6 mice, BAY 11-7082 alone had no effect on the expression of DC maturation molecules (CD80, CD86). Transforming growth factor beta (TGF-*β*), alone and in combination with BAY 11-7082, reduced the expression of these molecules. Cocultivation of DCs with splenocytes in the presence of TGF-*β* and BAY 11-7082 favored regulatory T cell (CD4+CD25hiFoxP3+) differentiation and disfavored differentiation of CD4+ T cells producing IL-10. In CBA mice, we found that rmIL-10 and rmTGF-*β* have a weak effect on maturation of DCs and their functional properties to induce Treg cells and IL-10 production.

**Conclusion:**

These results indicate that TGF-*β* and IL-10 have different effects on the phenotypic and functional characteristics of DCs and that the NF-*κ*B inhibitor, BAY 11-7082, has no synergistic effect on these treatments. In mice with an opposite nature of the immune response, the effects of immunoregulatory cytokines (IL-10 and TGF-b) differ on maturation of dendritic cells.

## 1. Introduction

The immune system mediates efficient protective responses against foreign pathogens but does not respond to self-antigens, thus preserving the integrity of the organism and maintaining immunological tolerance. This selective response to antigens depends on specific regulatory mechanisms, including regulatory immune cells. Under normal conditions, maintaining self-tolerance and controlling the intensity and duration of inflammatory immune responses are the primary functions of regulatory cells. However, forced induction of immunological tolerance is required under some conditions, such as autoimmune diseases or transplantation. Immunosuppressive drugs such as corticosteroids and cytotoxic drugs, which often cause systemic immunosuppression and thereby block the development of protective effector responses, are currently used to treat such diseases [1].

Cellular therapy approaches based on the use of tolerogenic cell populations, such as regulatory T cells (Tregs), are an alternative strategy for inducing immunological tolerance. The experiments in murine models have demonstrated that the presence of Tregs during skin or heart transplantation is critical for induction and maintenance of tolerance [2]. Furthermore, it was shown that simultaneous administration of purified Tregs and allografts during bone marrow transplantation significantly reduced graft-versus-host disease and improved engraftment **[** 3]. One obvious advantage of allospecific cellular therapies is that immunomodulation occurs only at the site of the alloantigen due to expression of immunosuppressive chemokines [4]. In this regard, previous studies have suggested that cell therapy is not associated with increased risk of infectious complications and cancer, as opposed to systemic immunosuppression associated with systemic drug administration [1].

The immunosuppressive properties of Tregs depend on the presentation of antigen by dendritic cells (DCs) during the immune response. *In vitro* experiments have shown that TCR-mediated activation is required to initiate the suppressive mechanisms of Tregs **[** 5]. Treg activation occurs in the periphery in an antigen-specific manner, as it is for all T cells. Additionally, the interaction between the CD28 coreceptor on Treg cells and B7-1 (CD80) and B7-2 (CD86) on DCs [6] is needed for activation. Thus, we assume that DCs expressing costimulatory molecules (CD80, CD86) and producing proinflammatory cytokines are required for induction of Tregs.

The main purpose of this study was to evaluate the effect of IL-10 and TGF-*β* and their combinations with BAY 11-7082. Such studies have neither been conducted nor published previously. IL-10 and TGF-*β* are known to affect the differentiation and functional characteristics of animal (mouse and human) dendritic cells; however, no studies have been performed for the combination with NF-*κ*B inhibitor (BAY 11-7082). The effect of BAY 11-7082 on various characteristics of dendritic cells has also been shown earlier. Meanwhile, it was interesting to study whether the synergistic effect is observed when using immunosuppressive cytokines and BAY 11-7082, since the effects of cytokines are realized (including through NF-*κ*B). This hypothesis was tested in this study.

IL-10, TGF-b, and BAY 11-7082 are known to have a suppressor effect on immune cells; these studies were performed using the same mouse line or human cells. It is important to know and understand that induction of tolerogenic dendritic cells depends not only on a specific suppressor facto but also on the general reactivity of the organism. Therefore, comparative studies need to be conducted in different lines of mice with opposite properties.

So, the mouse lines C57Bl/6 and CBA are opposed by a number of characteristics.

A number of scholars have previously demonstrated that C57Bl/6 and CBA mice have opposite properties. The differences consisted in varied reactivity of the immune system, the system of mononuclear phagocytes, and their regulation [7]. Furthermore, the responses of the immune and hormonal systems of CBA and C57Bl/6 mice to bacterial and fungal agents, *M. tuberculosis* [8] and *C. albicans* [1], were also different. CBA and C57Bl/6 mice are opposite in a number of ways: according to such properties as activity of metabolic processes in the liver [9], sensitivity to infectious agents [10, 11], structural and functional characteristics of the adrenal cortex [9], quantitative representation of cells of the mononuclear phagocyte system in various organs [12], and activation of immune cells [7]. According to the previously described interline differences, it can be assumed that mammals with different genetic programs have different ways of responding to the immune system. Therefore, this study was aimed at studying the characteristics of the immune response in the opposite CBA and C57Bl/6 mice.

This study is aimed at evaluating the effects of various immunosuppressive factors (IL-10, TGF-*β*, the NF-*κ*B inhibitor BAY 11-7082, and combinations thereof) on the phenotypic and functional characteristics of primary dendritic cells from C57BL/6 and CBA mice *in vitro*.

## 2. Materials and Methods

### 2.1. Ethics Statement

All the procedures performed in the experiments involving animals complied with the ethical standards of the institution. All the experimental protocols and methods were approved by the Institutional Review Board of the Research Institute of Fundamental and Clinical Immunology, Novosibirsk, Russian Federation (Protocol No. 99/2016-02-09). The study followed the principles outlined in the Declaration of Helsinki for all human or animal experiments.

### 2.2. Animals

Two-month-old female C57BL/6 and CBA mice (body weight, 20–25 g) were obtained from the breeding facility of the Institute of Cytology and Genetics (Novosibirsk, Russia). The animals were housed in the animal facility at the Research Institute of Fundamental and Clinical Immunology under natural light conditions with *ad libitum* access to food and water.

### 2.3. Media and Reagents

The commercial sources of cell culture reagents were as follows: RPMI 1640 culture media (BioloT), serum-free and phenol-red-free Opti-MEM (Thermo Fisher Scientific), fetal calf serum (FCS; HyClone), 2-mercaptoethanol (Sigma), L-glutamine (BioloT), bovine insulin (Pan-Eco), gentamicin (KRKA), benzylpenicillin (Sintez), HEPES buffer (Sigma), propidium iodide (PI) (Sigma), and 20% (*v*/*v*) Tween (Sigma). Monoclonal antibodies for DC phenotyping were purchased from BioLegend (CD11c-FITC, H2b-PE, H2k-PE, CD86-APC-Cy7, CD80-Brilliant Violet 450, CD4-PerCP, CD25-APC, IL-10-PE, and FoxP3-PE). Recombinant murine cytokines (IL-4, GM-CSF) were procured from R&D Systems. The mouse IL-10 quantitation immunoassay (Quantikine ELISA) was procured from R&D Systems. The colorimetric PreMix WST-1 assay to assess cell proliferation was purchased from Takara (Japan). Culture flasks and plates were from TPP (Switzerland), Petri dishes were from Nunclon, and monensin and brefeldin A were from Sigma.

### 2.4. Isolation of Bone Marrow Cells (BMCs)

Bone marrow was harvested from mouse femurs. The harvested cell suspension was washed twice with RPMI 1640 and centrifuged at 300*g* for 10 min. The adherent fraction of BMCs was obtained by plating in Petri dishes (maximum density, 20 million cells per dish) and incubating for 30 min at 37°C in an atmosphere of 5% CO_2_. The medium containing nonadherent cells was removed. The Petri dish was washed with RPMI 1640. Cells were then harvested by scraping and were precipitated by centrifugation at 300*g* for 10 min. The total number of cells in the adherent BMC fraction was determined using a Beckman Coulter blood analyzer and normalized to 10^6^ cells per mL.

### 2.5. Induction of DCs from Adherent BMC Fractions

Adherent BMC fractions were cultured in 48-well plates at a concentration of 10^6^ cells per mL of RPMI 1640 supplemented with 10% (*v*/*v*) FCS, 2 mM L-glutamine, 10 mM HEPES buffer, 0.5 mM 2-mercaptoethanol, 80 *μ*g/mL gentamicin, 100 *μ*g/mL penicillin, 20 ng/mL GM-CSF, and 20 ng/mL IL-4. On day 2 of DC cultivation, the culture medium was changed; 20 ng/mL GM-CSF and 20 ng/mL IL-4 were added again without cell sedimentation to increase the yield of DCs. Study groups were formed three days after culture initiation. The following immunosuppressive molecules were added to the cells: BAY 11-7082 (2.5 *μ*M), IL-10 (10 ng/mL), and TGF-*β* (10 ng/mL). Immature DCs cultured in the presence of GM-CSF and IL-4 alone were used as controls.

Six days after culture initiation, 2 × 10^5^ cells were selected for phenotypic analysis. The remaining DCs were precipitated by centrifugation and diluted with a complete medium to a concentration of 10^6^ per mL to be subsequently cocultured with splenocytes.

### 2.6. Coculturing of Splenocytes and DCs to Evaluate the Tolerogenic Properties of DCs

The functional properties of DCs were evaluated by coculturing with splenocytes. Allogeneic cells of the respective mouse lines (C57Bl/6 and CBA) were used for cocultivation of induced dendritic cells and splenocytes. Spleens of C57BL/6 and CBA mice were separated from the surrounding tissues and subjected to mechanical disaggregation. Сell suspensions were washed twice with RPMI 1640 and centrifuged at 300*g* for 10 min. The cells were resuspended and incubated with DCs at a ratio of 10 : 1 (total density, 10^6^ cells per mL) in the culture medium in a 48-well plate for 96 h. A monoculture of purified splenocytes cultured under the same conditions was used as a control.

### 2.7. Evaluation of Phenotypic Characteristics of DCs

The phenotypic and functional characteristics of DCs were evaluated on a BD FACSVerse flow cytometer after labeling using appropriate combinations of monoclonal antibodies. After staining, cells were washed in 500 *μ*L of PBS containing azide and fixed in 200 *μ*L of cold 1% paraformaldehyde solution for subsequent analysis.

### 2.8. Measurement of the Frequency of Tregs and Intracellular Expression of IL-10 and TGF-*β* in the Cocultures of Splenocytes and DCs

The functional characteristics of DCs were evaluated according to the frequency of CD4+CD25^hi^FoxP3+ Tregs, as well as intracellular expression of the immunoregulatory cytokines IL-10 and TGF-*β* in CD4+ T lymphocytes from the cocultures of splenocytes and DCs. For this experiment, 200 *μ*L of cells was added to a 96-well plate; monensin (10 *μ*M/mL) and brefeldin A (10 *μ*g/mL) were then added. The mixture was gently mixed and cultured for 3 h at 37°C in an atmosphere containing 5% CO_2_. Cell staining was carried out using monoclonal antibodies against CD4 and CD25 according to the manufacturer's protocol. For intracellular staining, cells were fixed with 1% paraformaldehyde, permeabilized with 0.1% saponin solution, and stained with monoclonal antibodies against IL-10, TGF-*β*, or FoxP3. After incubation, cells were washed and fixed in 1% paraformaldehyde solution for subsequent analyses. Cell cultures without the addition of monoclonal antibodies were used as a control.

### 2.9. Assessment of IL-10 Level in Conditioned Media of the Coculture of Splenocytes and DCs

The level of the immunoregulatory IL-10 cytokine in conditioned media of splenocyte and DC cocultures was evaluated by ELISA using R&D commercial test kits according to the manufacturer's protocol. The optical density of the samples was measured in the dual-wavelength mode: using a 450 nm filter of the 540 nm reference filter. The cytokine level was determined according to the curve plotted using optical densities of the known cytokine amounts.

### 2.10. Evaluation of Cell Proliferation in the Cocultures of Splenocytes and Tolerogenic DC

Proliferation of the coculture of splenocytes and DCs was evaluated using the PreMix WST-1 nonradioactive test according to the manufacturer's protocol. The test is based on quantitative measurement of formazan dye, whose amount increases in cultures of metabolically active cells. For this experiment, 100 *μ*L of cells (5 × 10^5^ cells per mL) was added to each well of a 96-well plate in three replicates; 10 *μ*L of PreMix WST-1 solution per well was added, and the plate was incubated for 4 h at 37°C in an atmosphere of 5% CO_2_. Splenocytes incubated under the same conditions were used as a control. Optical density at 440 nm was measured on a spectrophotometer. Proliferation was calculated as the ratio between the optical density of the samples containing mixed DCs and splenocytes and the optical density of splenocytes, with the media background subtracted.

### 2.11. Statistical Analyses

Statistical analyses were carried out using GraphPad Prism 6.0 software. The differences between the repeated measurements were assessed by ANOVA. Differences between the parameters were considered statistically significant at *p* < 0.05. All the data are shown as medians and interquartile ranges for nonnormally distributed data.

## 3. Results

### 3.1. Effects of the Tolerogenic Factors on Phenotypic Characteristics of DCs

In this study, GM-CSF and IL-4 were used for DC induction. The following tolerogenic factors were tested in this study: BAY 11-7082 (NF-*κ*B inhibitor) and the recombinant murine immunoregulatory cytokines IL-10 and TGF-*β*.

DCs are the major bone marrow-derived antigen-presenting cells. Antigens are captured, processed, and presented on the DC surface by MHC molecules (H-2b+ in C57BL/6 mice, H-2k+ in CBA mice). It is known that the expression of MHC molecules required for induction of regulatory cells is lower than that needed for induction of effector cells [5, 6].

**Figure 1:**
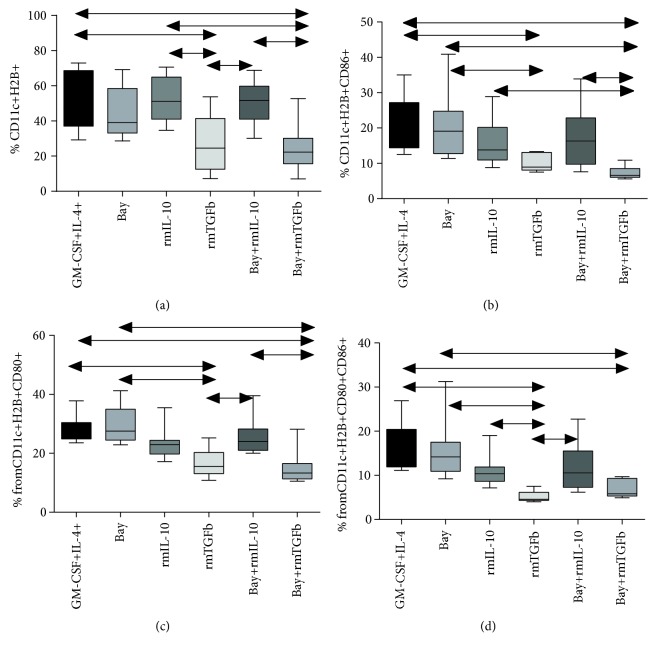
Expression of costimulatory molecules on bone marrow-derived DCs obtained from C57BL/6 mice. (a) Expression of H-2b on CD11c+ DCs. (b) Expression of CD86 on CD11c+H-2b+ DCs. (c) Expression of CD80 on CD11c+H-2b+DCs. (d) Expression of CD80/86 on CD11c+H-2b+ DCs. *N* = 18 mice per group. At least three independent experiments were performed. The arrows show significant intergroup differences (*p* < 0.05). Notes: GM-CSF+IL-4—BMCs cultured in the presence of GM-CSF and IL-4 alone; Bay—BMCs cultured in the presence of GM-CSF, IL-4, and BAY11-7082 added on day 2 of culturing; IL-10—BMCs cultured in the presence of GM-CSF, IL-4, and IL-10 added on day 3 of culturing; TGF-*β*—BMCs cultured in the presence of GM-CSF, IL-4, and TGF-*β* added on day 3 of culturing. Bay+IL-10—BMCs cultured in the presence of GM-CSF, IL-4, and BAY 11-7082 added on day 2 of culturing and IL-10 added on day 3 of culturing; Bay+TGF*β*—BMCs cultured in the presence of GM-CSF, IL-4, and BAY 11-7082 added on day 2 of culturing and TGF-*β* added on day 3 of culturing.

We demonstrated that treatment with BAY 11-7082 alone had no effect on the number of CD11c+ dendritic cells expressing H-2b+ in cultured C57Bl/6 BMCs (Figure 1(a)). In these cultures, the number of CD80+ and CD86+ DCs was not changed in the presence of these factors as compared with the number of DCs cultured in the absence of tolerogenic factors (immature DCs).

In the experiments with CBA mice, the amount of DCs generated from the bone marrow in the presence of rmGM-CSF and rmIL-4 was 2−2.5-fold lower than that in C57Bl6 mice. Addition of BAY 11-7082 had stronger pronounced toxic effects on cultured cells, leading to death of up to 70% of cells. For this reason, BAY 11-7082 was excluded from further research in CBA mice (data not shown).

Treatment with rmIL-10 alone or in combination with BAY 11-7082 had no effect on expression of MHC molecules on CD11c+ DCs in C57BL/6 BMC cultures (Figure 1(a)) as compared with either immature DCs or DCs cultured in the presence of BAY 11-7082 alone. Furthermore, cells cultured under these conditions exhibited no changes in the frequencies of CD80+ or CD86+ DCs (Figures 1(b)–1(d)).

**Figure 2:**
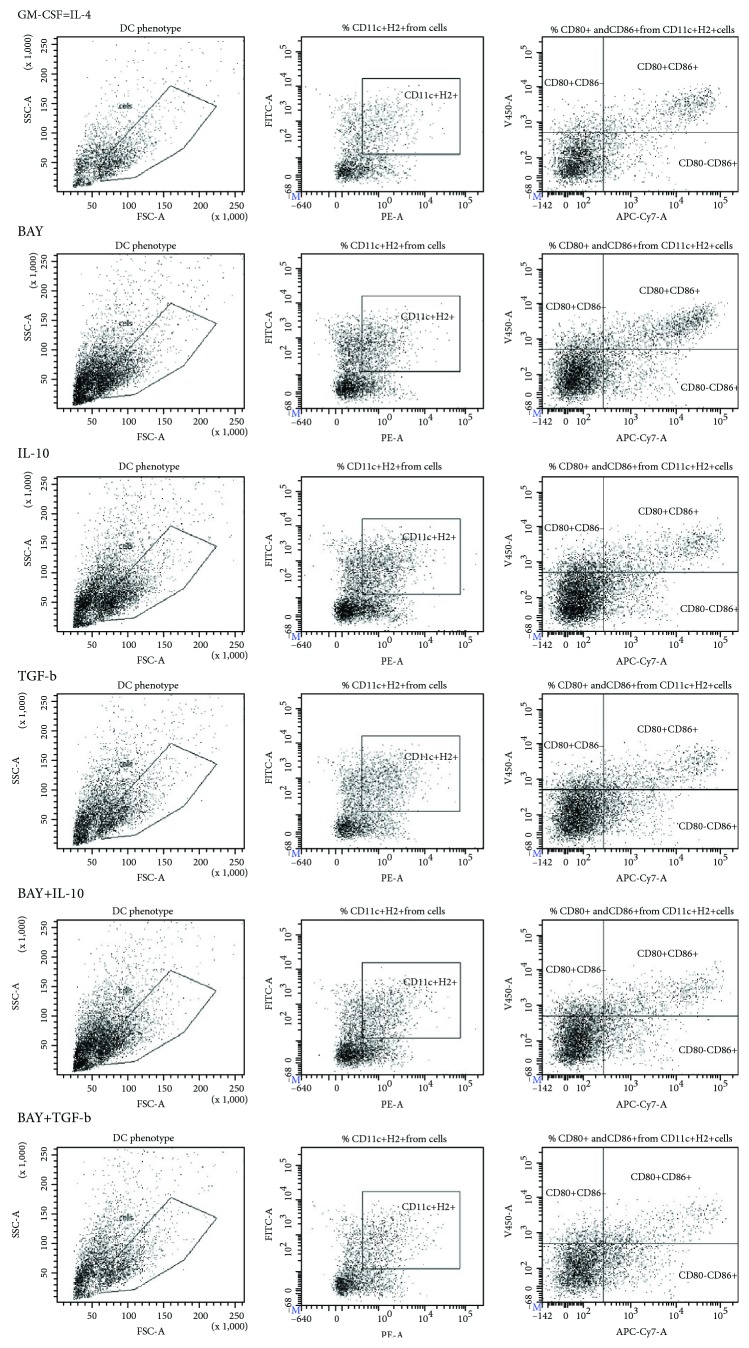
Expression of costimulatory molecules on bone marrow-derived DCs from C57BL/6 mice. (a) Expression of H-2b on CD11c+ DCs. (b) Expression of CD80/86 on CD11c+H-2b+ DCs.

In C57Bl6 mice, treatment with rmTGF-*β*, alone or in combination with BAY 11-7082, had the most significant effect on expression of H-2b on CD11c+ DCs. A significant decrease in H-2b expression in bone marrow-derived DCs was observed in these experimental groups (Figure 1) as compared with immature DCs or DCs exposed to other tolerogenic factors (rmIL-10, BAY 11-7082). The lowest frequencies of CD80+ and CD86+ DCs, as well as double-positive DCs, were observed in rmTGF-*β*-treated cultures. This result indicated that DC maturation in bone marrow cultures from C57BL/6 mice was significantly inhibited (Figures 1(b)–1(d)) under these conditions (Figures 2(a) and 2(b)).

**Figure 3:**
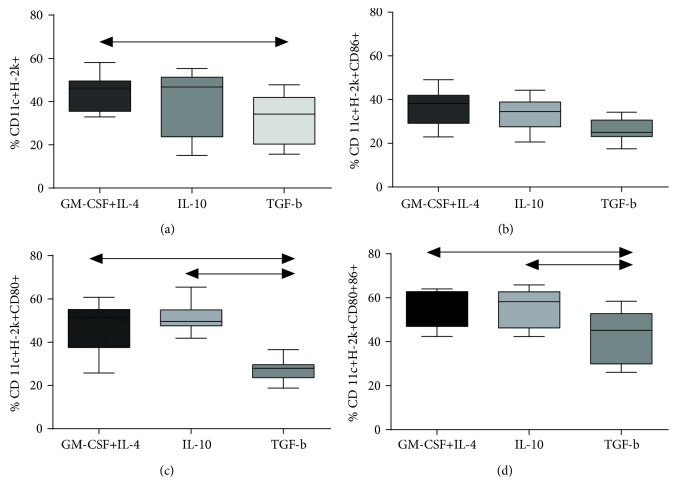
Expression of costimulatory molecules on bone marrow-derived DCs from CBA. (a) Expression of H-2K on CD11c+ DCs. (b) Expression of CD86 on CD11c+H-2k+ DCs. (c) Expression of CD80 on CD11c+H-2k+ DCs. (d) Expression of CD80/86 on CD11c+H-2k+ DCs. *N* = 18 mice per group. At least three independent experiments were performed. The arrows show significant intergroup differences (*p* < 0.05). Notes: GM-CSF+IL-4—BMCs cultured in the presence of GM-CSF and IL-4 alone; Bay—BMCs cultured in the presence of GM-CSF, IL-4, and BAY11-7082 added on day 2 of culturing; IL-10—BMCs cultured in the presence of GM-CSF, IL-4, and IL-10 added on day 3 of culturing; TGF-*β*—BMCs cultured in the presence of GM-CSF, IL-4, and TGF-*β* added on day 3 of culturing.

In CBA mice, cultivation of bone marrow cells in the presence of rmIL-10 neither affected the amount of CD11c+H-2K+ cells nor changed the expression of costimulatory markers (CD80, CD86) or their coexpression on the surface of differentiated DCs (Figure 2) regardless of the concentration of added rmIL-10. Addition of rmTGF-*β* reduced the amount of CD11c+H-2K+ cells and significantly reduced the expression of costimulatory molecules on the surface of DCs (CD80+, CD80+CD86+) as compared to the group of DCs with or without addition of rmIL-10 (Figure 3).

The results demonstrated that treatment with BAY 11-7082 (either alone or in combination with cytokines) did not affect the frequency of CD80+ and CD86+ C57BL/6 mice DCs but had pronounced toxic effects in CBA mice. rmTGF-*β* had a substantial inhibitory effect on the expression of MHC molecules and costimulatory molecules (CD80, CD86) on DCs, which is important for their tolerogenic ability.

### 3.2. Frequency of Tregs among Splenocytes Cocultured with Induced DCs

Tregs are the major population of regulatory cells responsible for natural and induced tolerance [10]. Tregs have the greatest suppressive ability, and the roles of other regulatory cells (DCs, B lymphocytes, and myeloid-derived suppressor cells) are indirectly related to induction and maintenance of Treg function [13]. Unlike the very heterogeneous population of Tregs in humans, activated CD4+CD25+ T cells expressing FoxP3 are believed to be the major population of Treg cells in mice [14].

**Figure 4:**
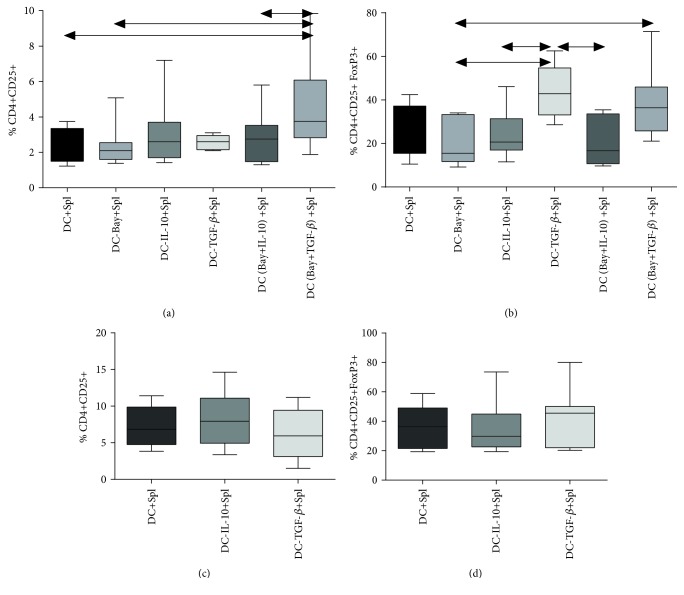
Expression of Treg markers in the cocultures of splenocytes and DCs from C57BL/6 and CBA mice. (a) The relative frequency of CD4+CD25+ cells in C57Bl/6 mice. (b) The relative frequency of FoxP3+ cells within CD4+CD25+ cells in C57Bl/6 mice. (c) The relative frequency of CD4+CD25+ cells in CBA mice. (d) The relative frequency of FoxP3+ cells in the CD4+CD25+ population in CBA mice. *N* = 18 mice per group. At least three independent experiments were performed. The arrows show significant intergroup differences (*p* < 0.05). Note: DC+Spl—coculture of immature DCs and splenocytes; DC-Bay+Spl—coculture of DCs treated with BAY 11-7082 and splenocytes; DC-IL-10+Spl—coculture of DCs treated with IL-10 and splenocytes; DC-TGF-*β*+Spl—coculture of DCs treated TGF-*β* and splenocytes; DC (Bay+IL-10)+Spl—coculture of DCs treated with BAY 11-7082 and IL-10 and splenocytes; DC (Bay+TGF*β*)+Spl—cocultures of DCs treated with a combination of BAY 11-7082 and TGF-*β* and splenocytes.

Since the DCs cultured in the presence of TGF-*β* (either alone or in combination with BAY 11-7082) had similar phenotypes, we assumed that both of these treatments had comparable effects on Treg induction in C57Bl/6 splenocytes. However, DCs treated with a combination of TGF-*β* and BAY 11-7082 showed a 1.5-fold increase in the amount of activated CD4+CD25hi cells in splenocyte-DC cocultures as compared with other experimental groups (Figure 4(a)). A two-fold increase in intracellular expression of FoxP3 was observed in the population of activated CD4+CD25hi lymphocytes (Figure 4(b)). Addition of rmTGF-*β* to the DC culture did not affect the frequency of activated lymphocytes in the splenocytes. However, a twofold increase in the frequency of CD4+CD25hiFoxP3+ Treg cells was observed. The use of other groups of DCs had no effect on the frequency of activated lymphocytes and expression of FoxP3, which was consistent with the phenotypic characteristics of the investigated DCs.

Investigation of the functional potential of generated tolDCs in CBA mice revealed that the amount of activated CD4+CD25hi cells and FoxP3+ Treg cells remained unchanged in DCs treated by rmIL-10 or rmTGF-*β* (Figures 3(c) and 3(d)).

Hence, we showed that cocultures of splenocytes and DCs treated with rmTGF-*β*, alone or in combination with BAY 11-7082, significantly increased the frequency of Tregs in C57Bl/6 mice. However, bone marrow cells obtained from CBA mice treated with rmTGF-*β* changed the phenotypic characteristics towards DCs but did not affect Treg induction.

### 3.3. The Frequency of CD4+IL-10+ Cells in Cocultures of Splenocytes and Induced DCs

Cytokine-mediated immunosuppression is one of the mechanisms of Treg regulatory function. IL-10 and TGF-*β* are believed to play the major role in Treg immunosuppression [15].

**Figure 5:**
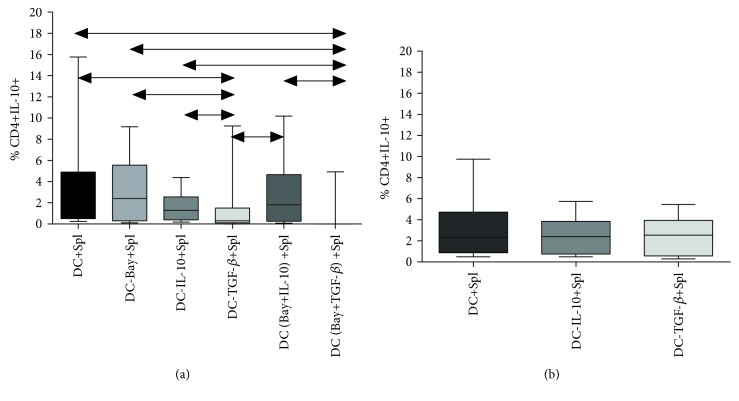
The frequency of IL-10-producing cells among CD4+ lymphocytes in the coculture of splenocytes and DCs in (a) C57Bl/6 mice and (b) CBA mice. *N* = 18 mice per group. At least three independent experiments were performed. The arrows show significant intergroup differences (*p* < 0.05, *N* = 18). Note: DC+Spl—coculture of immature DCs and splenocytes; DC-Bay+Spl—coculture of DCs treated with BAY 11-7082 and splenocytes; DC-IL-10+Spl—coculture of DCs treated with IL-10 and splenocytes; DC-TGF-*β*+Spl—coculture of DCs treated with TGF-*β* and splenocytes; DC (Bay+IL-10)+Spl—coculture of DCs treated with BAY 11-7082 and IL-10 and splenocytes; DC (Bay+TGF*β*)+Spl—coculture of DCs treated with BAY 11-7082 and TGF-*β* and splenocytes.

We found that the frequency of IL-10-producing CD4+ cells in C57Bl/6 mice decreased in the cocultures of splenocytes and DCs treated with TGF-*β*, as compared to the control group (GM-CSF- and IL-4-treated DCs) and the other experimental groups (DCs treated with BAY 11-7082 or IL-10) (Figure 5(a)). The intracellular production of IL-10 in CBA mice remained unchanged under these conditions (Figure 5(b)). We found no significant differences in frequency of TGF-*β*-positive CD4+ cells between the experimental groups in both mouse strains (data not shown).

We hypothesized that low expression levels of intracellular IL-10 may be related to its rapid secretion. To this end, we assessed the production of cytokines by cocultures of splenocytes and induced DCs.

### 3.4. IL-10 Level in Conditioned Media of the Cocultures of Splenocytes and DCs

**Figure 6:**
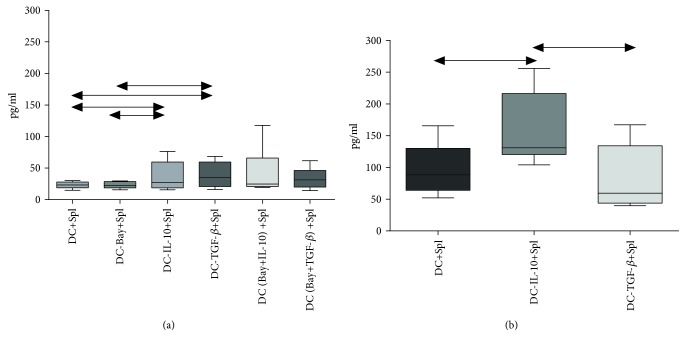
IL-10 level in the conditioned media of cocultures of splenocytes and induced tolerogenic DCs in (a) C57Bl/6 mice and (b) CBA mice. *N* = 18 mice per group. At least three independent experiments were performed. The arrows indicate significant intergroup differences (*p* < 0.05, *N* = 18). Note: DC+Spl—coculture of immature DCs and splenocytes; DC-Bay+Spl—coculture of DCs treated with BAY 11-7082 and splenocytes; DC-IL-10+Spl—coculture of DCs treated with IL-10 and splenocytes; DC-TGF-*β*+Spl—coculture of DCs treated with TGF-*β* and splenocytes; DC (Bay+IL-10)+Spl—coculture of DCs treated with BAY 11-7082 and IL-10 and splenocytes; DC (Bay+TGF*β*)+Spl—coculture of DCs treated with BAY 11-7082 and TGF-*β* and splenocytes.

We showed that DCs from C57Bl/6 mice treated with the tolerogenic factors TGF-*β* or IL-10 induced the tolerogenic potential of splenocytes, as opposed to immature DCs or DCs cultured in the presence of BAY 11-7082 (Figure 6(a)). The IL-10 level in the media of cocultures of splenocytes and DCs in the presence of rmIL-10 or rmTGF-*β* was significantly higher than that for immature DCs or DCs cultured in the presence of BAY 11-7082. Combining rmIL-10 or rmTGF-*β* with BAY 11-7082 during culturing of DC had no effect on IL-10 production by splenocyte cultures.

Investigation of the functional potential of generated tolDCs in CBA mice revealed that production of IL-10 was significantly increased in the coculture of splenocytes and DCs treated with rmIL-10. IL-10 production by CBA cell cultures was also 3-fold higher compared to that in C57Bl/6 cell cultures.

Hence, we have demonstrated that the number of CD4+ T cells containing intracellular IL-10 was reduced after coculturing of TGF-*β*-treated DCs with splenocytes but was associated with higher secretion of this cytokine in C57Bl6 mice. On the other hand, we have shown that intracellular production of IL-10 by CD4+ T cells in CBA mice remained unchanged. However, it was accompanied by elevated secretion of this cytokine.

### 3.5. Proliferative Activity of Splenocytes after Coculturing with Induced DCs

**Figure 7:**
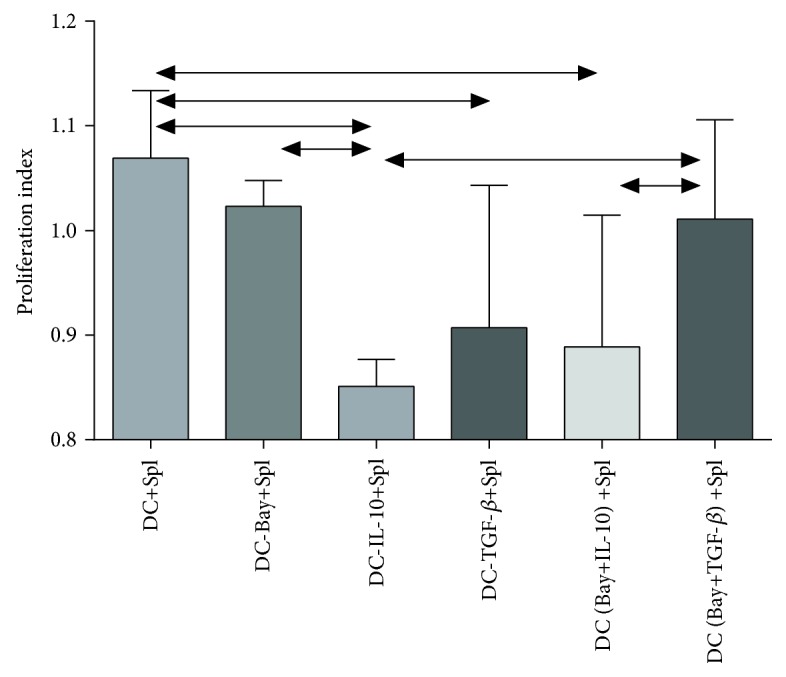
The proliferation index of the cocultures of splenocytes and DCs. The arrows indicate significant intergroup differences (*p* < 0.05, *N* = 18). Note: DC+Spl—coculture of immature DCs and splenocytes; DC-Bay+Spl—coculture of DCs treated with BAY 11-7082 and splenocytes; DC-IL-10+Spl—coculture of DCs treated with IL-10 and splenocytes; DC-TGF-*β*+Spl—coculture of DCs treated with TGF-*β* and splenocytes; DC (Bay+IL-10)+Spl—coculture of DCs treated with BAY 11-7082 and IL-10 and splenocytes; DC (Bay+TGF*β*)+Spl—coculture of DCs treated with BAY 11-7082 and TGF-*β* and splenocytes.

The lack of activation or inhibition of proliferation of splenocyte cultures is a characteristic effect of suppressor molecules on the surface of DCs. For this purpose, proliferative activity was assessed using a commercial kit according to the manufacturer's recommendations. The results are presented as relative units characterizing stimulation of proliferative activity of splenocytes in the presence of DCs. The effect of the environment on optical density was taken into account. In C57BL/6 mice, we found that DCs cultured in the presence of rmGM-CSF and rmIL-4 alone stimulated splenocyte proliferation (Figure 7). DCs cultured in the presence of rmIL-10, either alone or in combination with BAY 11-7082, were capable of maximum inhibition of splenocyte proliferation as compared with other experimental groups of DCs. DCs cultured in the presence of rmTGF-*β* tended to suppress splenocyte proliferation. Interestingly, addition of BAY 11-7082 to rmTGF-*β* during culturing of DCs had a negative effect on the tolerogenic properties of DCs. These DCs were unable to inhibit splenocyte proliferation. DC treated with BAY 11-7082 had no effect on proliferation of splenocyte cultures. These results demonstrate the immunosuppressive effects of DCs cultured in the presence rmTGF-*β* or rmIL-10 (either alone or in combination with BAY 11-7082) on proliferation of immunocompetent murine cells. In similar CBA cocultures, pretreated DCs had no effect on the proliferation index (data no shown).

Hence, we obtained ambiguous results on the influence of various suppressors on the phenotype and immunoregulatory properties of induced DCs. We found that the use of rmTGF-*β*, either alone or in combination with BAY 11-7082, increased the frequency of DCs with tolerogenic phenotypes in BMC cultures. These DCs were capable of inducing CD4+CD25+Foxp3+ Treg cells in splenocyte cocultures. However, these Treg cells used the mechanisms unrelated to cytokine production for tolerance induction. The use of other tolerogenic factors (BAY 11-7082, rmIL-10, and their combinations) did not significantly influence the immunoregulatory properties of induced DCs. These results demonstrate the effect of different immunosuppressive molecules on inhibition of induced maturation of dendritic cells from progenitor cells (BMCs).

It was shown that rmIL-10, unlike rmTGF-*β*, does not affect the phenotypic characteristics of DC of CBA mice compared to immature DC (the group with rmGM-CSF and rmIL-4). However, the observed decrease in phenotypic parameters of rmTGF-*β*-treated DCs did not influence the amount of CD4+CD25hiFoxP3+ T cells and IL-10 production.

## 4. Discussion

Throughout evolution, the immune system has developed effective mechanisms to control immune response against foreign antigens in the absence of response to self-antigens. However, there are some medical conditions (e.g., transplantation of organs and tissues) when unresponsiveness to antigens needs to be induced. The current studies dealing with this problem focus on the impact of regulatory factors on induction of various populations of tolerogenic cells. These studies have evaluated the effects of such molecules (transcription factors, costimulatory receptors, and cytokines) on modulation of pro- and/or anti-inflammatory cell functions. In this study, we have assessed the impact of three molecules (BAY 11-7082, rmIL-10, and rmTGF-*β*) on the key characteristics of induced DCs.

IL-10 and TGF-*β* are anti-inflammatory cytokines. These cytokines are natural regulators of immune processes in mammals due to expression of respective receptors on most hematopoietic cells [16, 17]. IL-10 inhibits IFN-*γ* production by acting on DCs, inhibiting the expression of costimulatory molecules (CD80, CD86) and antigen presentation to Th1 but not Th2 lymphocytes [18]. Coculturing of T cells with IL-10 results in inhibition of IL-2 and IFN-*γ* production and induces T suppressor/regulatory cells [19]. Considering the aforementioned facts, we have evaluated the influence of IL-10 on maturation of DCs induced in the presence of GM-CSF and IL-4 in the *in vitro* model system.

We showed that rmIL-10 added on day 3 of culturing of DCs in the presence of rmGM-CSF and rmIL-4 had a significant effect on expression of costimulatory molecules (CD80, CD86) on DCs in neither C57BL/6 nor CBA mice. For splenocyte cocultures, increased IL-10 level in the conditioned media and suppression of proliferative activity of splenocytes were observed as compared to DCs cultured without tolerogenic factors or in the presence of BAY 11-7082 alone. This fact may indicate that IL-10 modulates the functional status of DCs without suppressing their maturation.

TGF-*β* is a pleiotropic cytokine regulating proliferation, differentiation, migration, and apoptosis of various cell types; it acts as a negative regulator. TGF-*β* plays a key role in the function of all immune cells and in regulation of T cell development and induction of immunological tolerance involving DCs in particular. TGF-*β* enhances the expression of the FoxP3 transcription factor and differentiation of Treg cells, thereby facilitating the development of tolerance. Along with its influence on proliferation of cytotoxic cells, TGF-*β* suppresses perforin and granzyme gene expression. Perforin and granzyme are key proteins of the cytotoxic program of CD8+ T lymphocytes [20].

In this study, we demonstrated that rmTGF-*β* added to BMCs cultured in the presence of rmGM-CSF and rmIL-4 significantly reduced the expression of costimulatory molecules on the surface of CD11c+H2-b+ DCs in C57BL/6 mice. The coculture of splenocytes and DCs treated with rmTGF-*β* significantly increased the frequency of CD4+CD25+FoxP3+ Treg cells and IL-10 production. Furthermore, reduced proliferative activity of splenocytes was observed as compared to the group of immature DCs. These results are indicative of the pronounced influence of TGF-*β* on the tolerogenic capacity of cell cultures. Reduced intracellular expression of IL-10 in CD4+ splenocytes as compared with the control group and the significantly lower IL-10 level in the conditioned media of the cocultures of splenocytes and DCs were associated with increased IL-10 production and secretion by these cells.

In CBA mice, a similar culturing of BMC in the presence of rmTGF-*β* did not affect the amount of CD11c+H2K+ cells but significantly reduced the expression of costimulatory molecules on the surface of DCs (CD80, CD86+CD80+) as compared to the group of DCs with or without addition of rmIL-10. However, rmTGF-*β*-treated DCs affected neither the frequency of CD4+CD25+FoxP3+ Treg cells nor IL-10 production in CBA mice.

BAY 11-7082 is known to block the activation of NF-*κ*B by affecting I*κ*B phosphorylation [21]. In a cell culture, BAY 11-7082 inhibits a wide range of TNF-*α*-induced proinflammatory processes, including DC differentiation and maturation [22]. However, our study showed that BAY 11-7082 had virtually no effect on the phenotype and functional activity of induced DCs in C57Bl/6 mice. The absence of an inhibitory effect of BAY 11-7082 can be attributed to several factors. First, BMCs cultured in the presence of rmGM-CSF and rmIL-4 for 2 days were differentiated into immature DCs. Furthermore, BAY 11-7082 is a synthetic molecule that irreversibly inhibits only NF-*κ*B, not excluding the involvement of other transcriptional pathways in induction of DC maturation. BAY 11-7082 added to C57Bl/6 cell cultures at earlier stages and any stages of CBA cell culture (day 0 or 1) resulted in pronounced destruction of BMCs (Supplement 1). Therefore, we decided to test whether BAY 11-7082 and cytokines (IL-10 and TGF-*β*) exhibit a synergistic effect in C57Bl/6 mice due to the less pronounced toxic effect than they have in CBA mice. However, simultaneous application of BAY 11-7082 and rmIL-10 or BAY 11-7082 and rmTGF-*β* for DC differentiation showed no effect on expression of costimulatory molecules or the tolerogenic potential of splenocyte cultures. These data further support that BAY 11-7082 has no effect on the tolerogenic properties of BMC cultures.

Therefore, the results showed that the tolerogenic factor rmTGF-*β* alone induced DCs with tolerogenic properties regardless of whether BAY 11-7082 was present or not.

## 5. Conclusion

Our results demonstrate that the influence of immunosuppressive substances on the phenotypic and functional characteristics of induced DCs is heterogeneous, thus providing additional evidence for multifactorial regulation of DC differentiation and nonlinear development of the immune response. DC differentiation was affected not only by quantitative and qualitative characteristics of the regulatory factor but also by the development stage (differentiation or activation) of a target biological object (and DCs in particular).

## Data Availability

The data used to support the findings of this study are available from the corresponding author upon request.

## Supplementary Materials

Supplementary Materials The additional file contains figures with the results of the evaluation of the frequency of CD11c+H-2b+ cells depending on the timing of adding BAY 11-7082 to the culture of induced DC. It was shown that adding BAY 11-7082 on day 0 of cultivation of DС critically reduces the level of CD11c+H-2b+ cells, in contrast to adding BAY 11-7082 on day 2 of cultivation of DС.
