# Supplementary material for: The Effects of Immunosuppressive Factors on Primary Dendritic Cells from C57BL/6 and CBA Mice
Source: J Immunol Res. 2019 Apr 18;2019:7029726. doi: 10.1155/2019/7029726 (PMC6501430; doi:10.1155/2019/7029726)
Supplement: Supplementary Materials — The additional file contains figures with the results of the evaluation of the frequency of CD11c+H-2b+ cells depending on the timing of adding BAY 11-7082 to the culture of induced DC. It was shown that adding BAY 11-7082 on day 0 of cultivation of DС critically reduces the level of CD11c+H-2b+ cells, in contrast to adding BAY 11-7082 on day 2 of cultivation of DС. [file 7029726.f1.pdf]

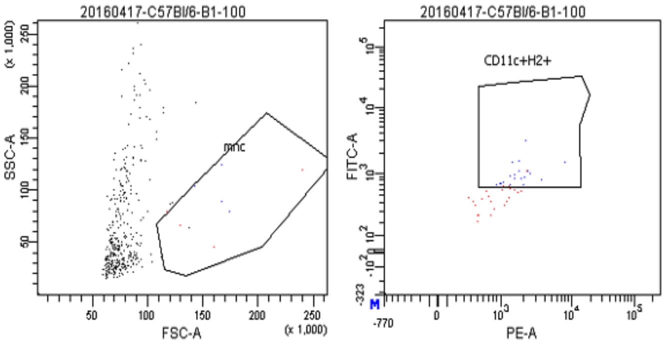

B.

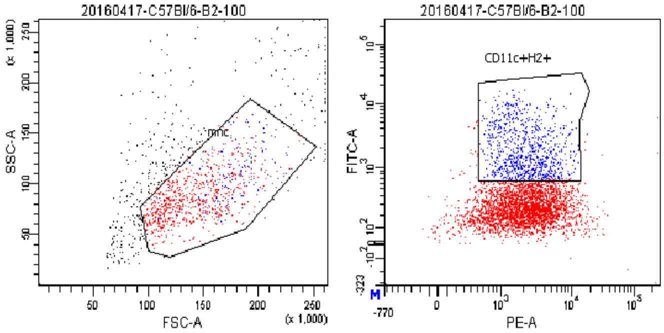

Supplement 1. Assessment of the effect of time of addition of BAY 11-7082 to cultures of induced DCs on the frequency of CD11c+H-2b+ cells.

Note: A — analysis of FSC and SSC distribution of events and the frequency of CD11c+H-2b+ bone marrow cells, when adding BAY 11-7082 on day 0 of culture in the presence of GM-CSF and IL-4; B — Analysis of FSC and SSC distribution of events and the content of CD11c+H-2b+ BMCs, when adding BAY 11-7082 on day 2 of culture in the presence of GM-CSF and IL-4.
